# Effectiveness and brain mechanism of multi-target transcranial alternating current stimulation (tACS) on motor learning in stroke patients: study protocol for a randomized controlled trial

**DOI:** 10.1186/s13063-024-07913-4

**Published:** 2024-01-30

**Authors:** Ming-Hui Lai, Xiao-Ming Yu, Yan Lu, Hong-Lin Wang, Wang Fu, Huan-Xia Zhou, Yuan-Li Li, Jun Hu, Jiayi Xia, Zekai Hu, Chun-Lei Shan, Feng Wang, Cong Wang

**Affiliations:** 1 Department of Rehabilitation, Seventh People’s Hospital of Shanghai University of Traditional Chinese Medicine, Datong Rd. 358, Shanghai, 200137 China; 2 Department of Neurology, Seventh People’s Hospital of Shanghai University of Traditional Chinese Medicine, Shanghai, 200137 China; 3 Engineering Research Center of Traditional Chinese Medicine Intelligent Rehabilitation, Ministry of Education, Shanghai, 201203 China; 4 School of Rehabilitation Science, Shanghai University of Traditional Chinese Medicine, Shanghai, 201203 China; 5 The Second Rehabilitation Hospital of Shanghai, Shanghai, 200435 China; 6 Queensland Brain Institute, the University of Queensland, Brisbane, 4072 Australia

**Keywords:** Transcranial alternating current stimulation, Motor learning, 40-Hz multi-target stimulation, Stroke rehabilitation

## Abstract

**Background:**

Transcranial alternating current stimulation (tACS) has proven to be an effective treatment for improving cognition, a crucial factor in motor learning. However, current studies are predominantly focused on the motor cortex, and the potential brain mechanisms responsible for the therapeutic effects are still unclear. Given the interconnected nature of motor learning within the brain network, we have proposed a novel approach known as multi-target tACS. This study aims to ascertain whether multi-target tACS is more effective than single-target stimulation in stroke patients and to further explore the potential underlying brain mechanisms by using techniques such as transcranial magnetic stimulation (TMS) and magnetic resonance imaging (MRI).

**Methods:**

This study employs a double-blind, sham-controlled, randomized controlled trial design with a 2-week intervention period. Both participants and outcome assessors will remain unaware of treatment allocation throughout the study. Thirty-nine stroke patients will be recruited and randomized into three distinct groups, including the sham tACS group (SS group), the single-target tACS group (ST group), and the multi-target tACS group (MT group), at a 1:1:1 ratio. The primary outcomes are series reaction time tests (SRTTs) combined with electroencephalograms (EEGs). The secondary outcomes include motor evoked potential (MEP), central motor conduction time (CMCT), short interval intracortical inhibition (SICI), intracortical facilitation (ICF), magnetic resonance imaging (MRI), Box and Block Test (BBT), and blood sample RNA sequencing. The tACS interventions for all three groups will be administered over a 2-week period, with outcome assessments conducted at baseline (T0) and 1 day (T1), 7 days (T2), and 14 days (T3) of the intervention phase.

**Discussion:**

The study’s findings will determine the potential of 40-Hz tACS to improve motor learning in stroke patients. Additionally, it will compare the effectiveness of multi-target and single-target approaches, shedding light on their respective improvement effects. Through the utilization of techniques such as TMS and MRI, the study aims to uncover the underlying brain mechanisms responsible for the therapeutic impact. Furthermore, the intervention has the potential to facilitate motor learning efficiency, thereby contributing to the advancement of future stroke rehabilitation treatment.

**Trial registration:**

Chinese Clinical Trial Registry ChiCTR2300073465. Registered on 11 July 2023.

## Administrative information

Note: the numbers in curly brackets in this protocol refer to SPIRIT checklist item numbers. The order of the items has been modified to group similar items (see http://www.equator-network.org/reporting-guidelines/spirit-2013-statement-defining-standard-protocol-items-for-clinical-trials/).

**Table:** 

Title {1}	Effectiveness and brain mechanism of multi-target transcranial alternating current stimulation (tACS) on motor learning in stroke patients: Study protocol for a randomized controlled trial
Trial registration {2a and 2b}.	Chinese Clinical Trial Registry ChiCTR2300073465 Please refer to Item 2a and registration in the Chinese Clinical trial register ChiCTR2300073465 https://www.chictr.org.cn/showprojEN.html?proj=199781
Protocol version {3}	Version 2 of Dec. 2023
Funding {4}	This study is supported by the National Natural Science Foundation of China (PI: Cong Wang, Grant No. 82202787), the Frontier Innovative Talents of Traditional Chinese Medicine Program of Shanghai University of Traditional Chinese Medicine (Grant No. 009), the Discipline Construction of Pudong Health Bureau of Shanghai (Grant No. PWZzb2022–11) and Xin Xing Talents Training Program of the Seventh People’s Hospital, Shanghai University of Traditional Chinese Medicine (Grant No. XX2023–06). The research equipment (multitarget tACS system, TMS, EEG recording and fMRI scanner) and clinical trial was supported by Shanghai Seventh People’s Hospital and Engineering Research Center of Traditional Chinese Medicine Intelligent Rehabilitation, Ministry of Education. This research is an investigator-initiated project and the funding sources had no role in the design of this study and will not have any role during its execution, analyses, interpretation of the data, or decision to submit results.
Author details {5a}	Ming-Hui Lai^1^, Xiao-Ming Yu ^1^, Yan Lu ^1^, Hong-Lin Wang^1^, Wang Fu^2^, Huan-Xia Zhou^1^, Yuan-Li Li^1,3,4^,Jun Hu^5^, Jiayi Xia^5^, Zekai Hu^5^, Chun-Lei Shan^1,3,4^, Feng Wang^2^, Cong Wang ^1,3,4,5,6^ 1 Department of Rehabilitation, Seventh People’s Hospital of Shanghai University of Traditional Chinese Medicine, Shanghai, 200,137, China 2 Department of Neurology, Seventh People’s Hospital of Shanghai University of Traditional Chinese Medicine, Shanghai, 200,137, China 3 Engineering Research Center of Traditional Chinese Medicine Intelligent Rehabilitation, Ministry of Education, Shanghai, 201,203, China 4 School of Rehabilitation Science, Shanghai University of Traditional Chinese Medicine, Shanghai, 201,203, China 5 The Second Rehabilitation Hospital of Shanghai, Shanghai, 200,435, China 6 Queensland Brain Institute, the University of Queensland, Brisbane, 4072, Australia
Name and contact information for the trial sponsor {5b}	Department of Rehabilitation, Seventh People’s Hospital of Shanghai University of Traditional Chinese Medicine, Datong Rd. 358, Shanghai 200,137, China. Phone: 86–21-58,611,047
Role of sponsor {5c}	The sponsor played no role in the study design, manuscript writing, data collection, management, analysis, and interpretation. The sponsor did not contribute to the decision to submit this report for publication and does not have ultimate authority over any of the authors’ activities.

## Introduction

### Background and rationale {6a}

Motor learning refers to the acquisition and solidification of a new motor skill, which plays an important role in rehabilitation activities [1]. The process of motor learning involves multiple brain regions, accompanied by changes in synaptic plasticity that can improve motor dysfunction caused by neurological damage, such as stroke [2]. In fact, the reacquisition of motor skills via motor learning holds the utmost significance in the recovery process of poststroke hemiparesis [3]. Therefore, the quest for efficacious tools and treatments to enhance motor learning after stroke becomes imperative.

Neural oscillations are rhythmic electric processes of neuron groups in the central nervous system that include five frequency bands: delta (δ, < 4 Hz), theta (θ, 4–7 Hz), alpha (α, 8–12 Hz), beta (β, 13–30 Hz), and gamma (γ, 30–100 Hz). In particular, γ oscillations are considered to emerge by activation of reciprocally connected excitatory pyramidal neurons and inhibitory interneurons and control the connectivity between different brain regions, which is crucial for perception, movement, memory, and emotion [4]. Studies show that γ oscillations, approximately 40 Hz, have been recorded during movement preparation during both externally triggered and self-paced hand movements [5], which means that γ oscillations are always present during movement onset and execution [6] and reflect the integration of visual, proprioceptive, and somatosensory information with ongoing motor activity.

Recent studies have shown that noninvasive brain stimulation (NIBS) techniques, such as transcranial alternating current stimulation (tACS), have emerged as potent tools to induce neural oscillations in the brain network. tACS can improve motor performance differentially by the injection of sinusoidal currents to modulate cortical excitability and brain electrical activity [7]. Compared to transcranial magnetic stimulation (TMS), tACS devices are inexpensive and convenient to use and have smaller electrode pads that can achieve multi-target stimulation in the brain. Furthermore, tACS can set different stimulation frequencies to induce different neural oscillation bands compared to transcranial direct current stimulation (tDCS).

A growing number of studies attest to the benefits of tACS in motor learning [8], motor skills enhancement [9], and cognitive augmentation [10]. Numerous studies concur that 40 Hz tACS significantly enhances cognitive function in healthy people and individuals with Alzheimer’s disease (AD) [11–16]. In addition, Hopfinger et al. revealed that 40-Hz tACS facilitates endogenous attention in mice with Alzheimer’s disease, highlighting the pivotal role of slow gamma oscillations (induced by 40 Hz stimulation) in attentional disengagement and reorientation [17]. The above studies reveal that the improvement of cognitive function could be achieved by 40-Hz tACS in both humans and mice. It is worth noting that the newest research affirms a significant correlation between cognitive ability and motor learning function in the elderly [18]. However, most current tACS studies focus on a single cortical stimulation area related to movement or cognition (single target), which neglects an important point of neural function regulated by brain networks [19–21]. Direct evidence regarding the impact and brain mechanism of multi-target 40 Hz tACS on the motor learning of stroke patients is currently lacking.

Many clinical studies have confirmed the functional connectivity between the primary motor cortex (M1), primary sensory cortex (S1), and dorsolateral prefrontal cortex (DLPFC). The coactivation of M1 and S1 and DLPFC in planning, execution, and control of movement has been well established [22–24]. In addition to the three brain regions mentioned above, recent studies have shown that cerebellar (CB) tACS can lead to improved cortical excitability and motor behavior [25], which affects balance and gait, limb movement, oculomotor control, and cognition.

## Objectives {7}

Based on the above theory, we proposed that simultaneous stimulation of M1, S1, DLPFC, and CB (multi-target tACS) can be more effective than single-target stimulation due to the enhancement of motor learning-related brain network interactions. With appropriate control experiments, the potential underlying brain mechanism will be studied by behavioral tasks and assessments of scales, EEG, MRI, and TMS. Furthermore, we will perform RNA sequencing analysis on the blood samples of the stroke participants before and after the intervention to further analyze the changes in neuroinflammatory factors, nerve growth factors, and synaptic plasticity genes. The objectives of this study are to explore the effects of multi-target tACS on motor learning function in stroke patients and explore the underlying brain mechanism.

## Trial design {8}

**Fig. 1:**
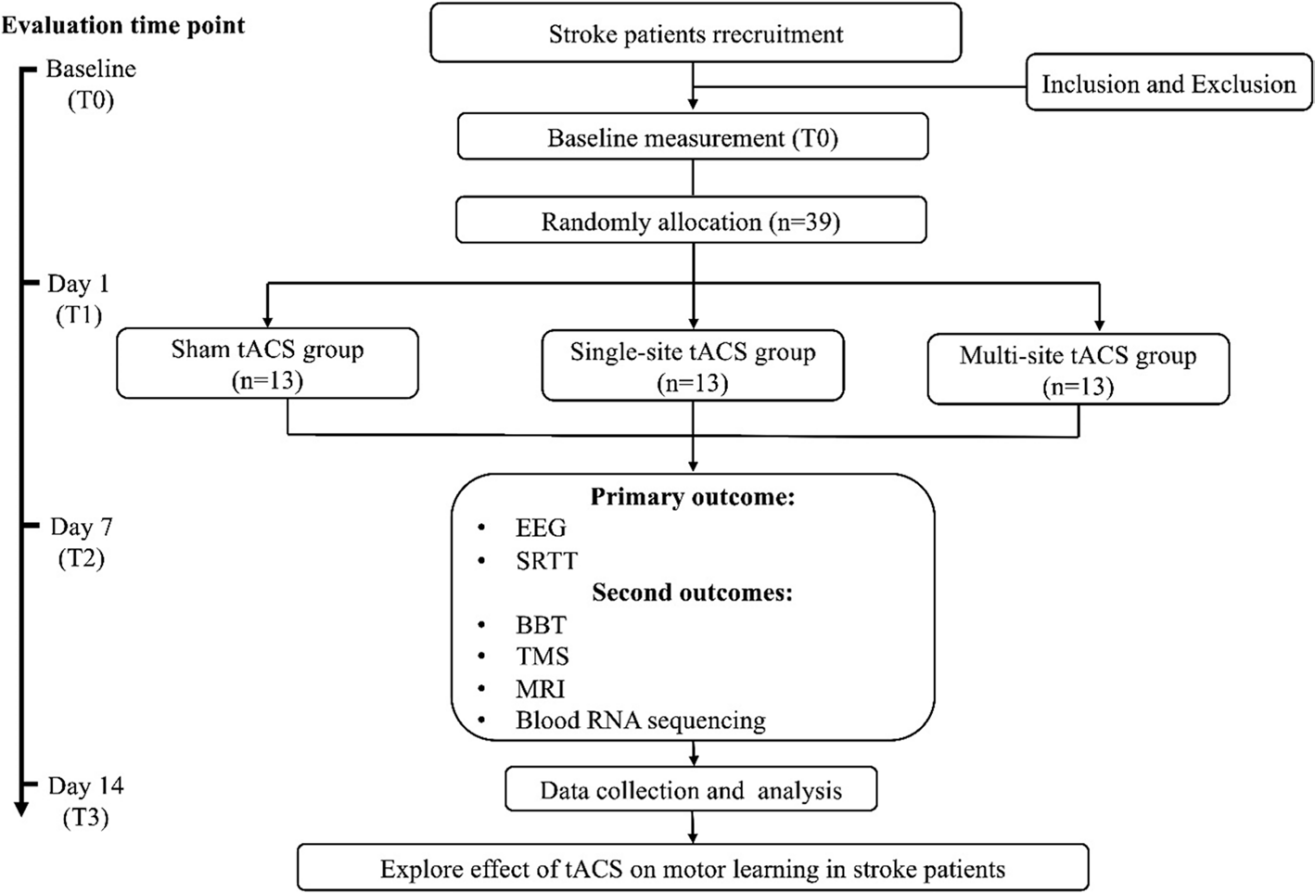
Comprehensive study flowchart of the protocol

This study will be a randomized controlled trial utilizing a parallel-group design. A total of 39 stroke patients will be assigned to three groups: the sham tACS group (SS group), single-target tACS group (ST group), and multi-target tACS group (MT group) at a 1:1:1 ratio as determined by a simple random sampling process. The comprehensive study flowchart is depicted in Fig. 1, while the study schedule is outlined in Table 1. The protocol was approved by the Ethics Committee of Shanghai Seventh People’s Hospital (2023-7th-HIRB-043) and will be conducted in accordance with the ethical standards of the Declaration of Helsinki. The protocol has been registered in the Chinese Clinical Trial Registry (ChiCTR2300073465) and used the SPIRIT reporting guidelines [26]. Prior to participating in the experiment, patients will be required to provide informed consent.

**Table 1:**
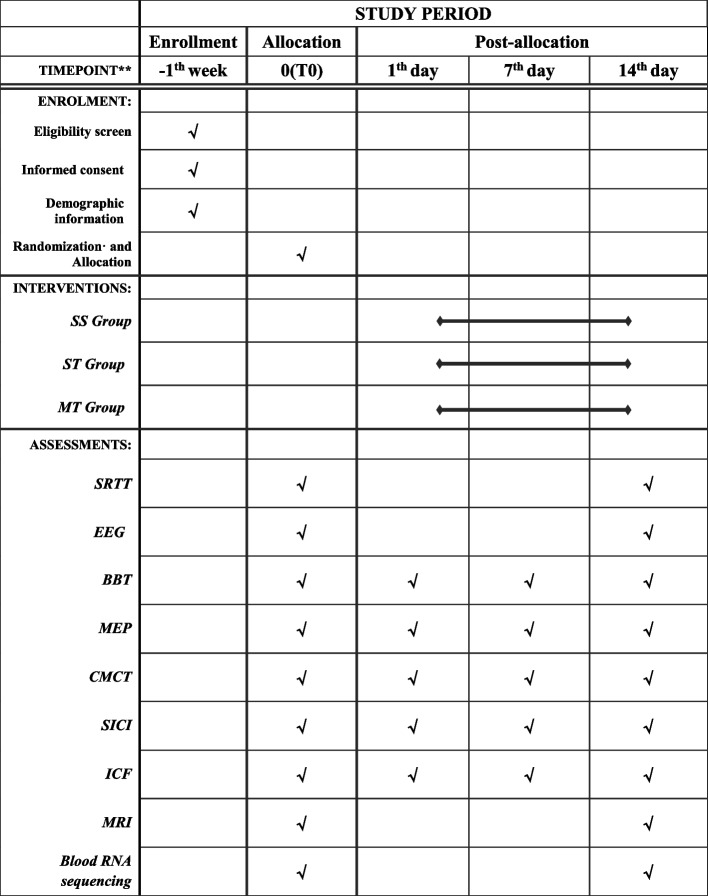
Schedule of enrollment, intervention, and assessments

“√” means things will be done. *SRTT* series reaction time test, *EEG* electroencephalogram, *BBT* Box and Block Test, *MEP* motor evoked potential, *CMCT* central motor conduction time, *SICI* short interval intracortical inhibition, *ICF* intracortical facilitation, *MRI* magnetic resonance imaging

## Methods: participants, interventions, and outcomes

### Study setting {9}

Recruitment of stroke patients, assessment of scales and equipment, and electrical stimulation interventions will all be conducted at the Shanghai Seventh People’s Hospital in China.

### Eligibility criteria {10}

#### Inclusion criteria

1. Age between 40 and 75 years, possessing normal or corrected-to-normal vision.
2. Right-handedness, as indicated by a score greater than 40 points on the Edinburgh Handedness Scale [27].
3. Experienced a single (first ever), unilateral stroke (ischemic or intracerebral hemorrhagic) within the timeframe of 2 weeks to 6 months from onset.
4. Exhibiting clear consciousness and absence of mental disorders, with a score exceeding 23 points on the Mini-Mental State Examination (MMSE) [28].
5. Demonstrating a willingness to cooperate in the research and providing voluntary consent by signing the informed consent form.
6. Presenting an upper limb with a Brunnstrom scale grade ranging from III to V.

#### Exclusion criteria

1. Presence of contradictions for tACS, TMS, or MRI, such as implantable electronic device or compromised skin in the stimulation area resulting from damage or hyperalgesia.
2. Diagnosis of any other neurological conditions, including depression, severe language comprehension deficits, unilateral spatial neglect, epilepsy, upper-limb apraxia, etc.
3. Proficiency in demanding finger movements, such as piano players, professional game players, and individuals with prior experience in sequential response time testing (SRTT).
4. Ongoing participation in concurrent clinical studies.
5. Identifications of additional reasons, as deemed by researchers, rendering the participant unsuitable for inclusion in the experiment.
6. Inability to comprehend or provide a signature on the informed consent form.

### Who will take informed consent? {26a}

The informed consent will be conducted by experienced neurologists and signed by two sides (blinded physicians and stroke patients). The potential participant has sufficient time to read the full text of the informed consent form, and neurologists will introduce the details of the contents of the informed consent form to them. Finally, the patient will decide whether to participate in the study by themselves. All participants will sign informed consent before the study, and all methods will be performed in accordance with the Declaration of Helsinki.

### Additional consent provisions for collection and use of participant data and biological specimens {26b}

After the patient signs informed consent, blood will be sampled by venipuncture of the elbow from participants. The entire blood sampling process is operated by a professional neurology nurse and extracts about 2 ml.

### Interventions

#### Explanation for the choice of comparators {6b}

The study included one sham and two active stimulation interventions delivered to three parallel groups.

In the SS group, a “pseudo stimulus” button will be discreetly activated, providing a semblance of stimulation to the subject without their awareness. For the ST group, the current channel will solely target M1, while the remaining three brain areas will receive pseudo stimulation, akin to the SS group. In the MT group, all four channels will be activated simultaneously. The entire process of electrical stimulation intervention will be closely supervised by a proficient clinical operator in conjunction with emergency personnel. Sham stimulation enables a placebo comparator to the active conditions.

#### Intervention description {11a}

The multi-target tACS will be performed using a multichannel transcranial electrical stimulator (YingChi, Shenzhen). All participants will undergo a daily 20-min tACS session over a 2-week period. A skilled physiotherapist will conduct the sham tACS, single-target tACS, or multi-target tACS intervention on patients under the standardized condition. The tACS session will be delivered to the cortex via surface sponge electrodes (5 × 7 cm) soaked in 0.9% NaCl, which will be employed and secured in place using elastic gauze. Based on previous experimental research [29], the following stimulation parameters will be employed: a stimulating intensity of 1 mA peak to peak and a stimulation frequency of 40 Hz. For electrode placement, participants will receive a total of 4 pairs of electrode pads. The anodes will be placed in the M1, S1, DLPFC, and CB in alignment with the affected brain area, while cathodes will be placed in the contralateral brain area for each corresponding electrode [30].

#### Criteria for discontinuing or modifying allocated interventions {11b}

There are no anticipated problems that are detrimental to the participant.

#### Strategies to improve adherence to interventions {11c}

To ensure the protocol adherence of the intervention and assessment, all the neurologists, physiotherapists, and nurses involved in the research project will be trained according to the recommended guidelines and protocols.

#### Relevant concomitant care permitted or prohibited during the trial {11d}

During the study, the stroke patients will be able to continue their daily treatment plan at the hospital as usual.

#### Provisions for post-trial care {30}

There is no anticipated harm for tACS intervention. However, participants who feel uncomfortable, such as skin itching, will be evaluated by the neurologist for further necessary treatment.

### Outcomes {12}

Assessments will be conducted by physiotherapists blinded to the group allocation at various designated time points, as outlined in Table 1. Additionally, a comprehensive questionnaire (administered during week 0) will capture baseline information, including age, gender, symptoms, disease severity, duration, prior treatment, and medication history, which will be recorded using a questionnaire. Additionally, any encountered side effects throughout the course of the study will be promptly documented and reported in real time.

#### Primary outcomes

##### Series reaction time test task (SRTT)

**Fig. 2:**
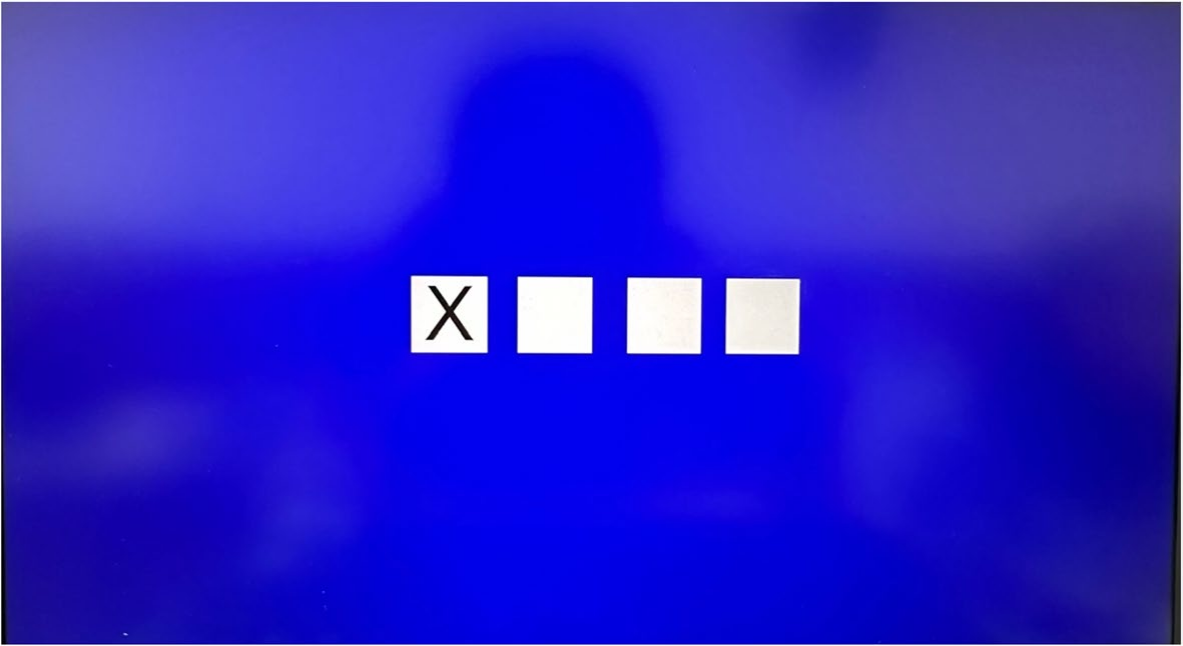
Screenshot of the SRTT software used in this study

The series reaction time task (SRTT) is the most commonly used method to assess motor learning function. The combination of SRTT and EEG is an important method for investigating the effects and brain mechanisms of motor learning [31]. We will use software (Deary-Liewald, UK) [32] to evaluate the changes in motor learning pre- and post-tACS intervention. Prior to commencing the SRTT, participants will be seated in front of a 15.6-in. computer screen with their hemiplegic-side hand resting on the keyboard. The mapping of fingers to the correspondence numbers is as follows: index finger = V, middle finger = C, ring finger = X, and little finger = Z. Four white squares will be positioned approximately in the center of a computer screen, contrasted against a blue background (refer to Fig. 2). The arrangement of the keyboard keys mirrors the layout of the squares on the screen: the “V” key aligns with the far-left square, “X” key with the second-left square, “C” key with the second-right square, and “Z” key with the far-right square. The task involves responding to the appearance of a diagonal cross within each square by promptly using the corresponding keyboard key. Participants are required to execute this key as quickly as possible upon each cross’s appearance.

The task includes 10 practice trials followed by 80 experiment trials, collectively lasting no more than 10 min for each participant. The interstimulus interval (measured in milliseconds, refers to the duration between a response and the subsequent appearance of a cross) will be randomly set within the range of 1000–3000 ms. The computer program will record the response time and the interstimulus interval for each trial [33].

##### Electroencephalogram (EEG)

**Fig. 3:**
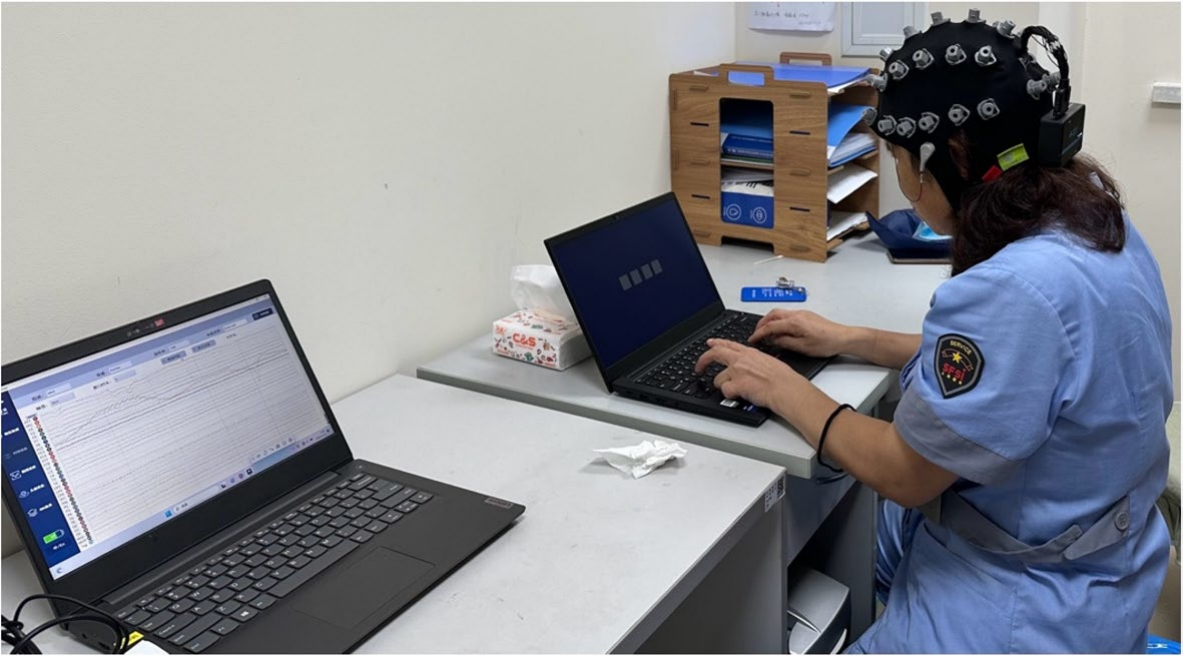
EEG will be recorded from the subject while performing the SSRT

A wireless multichannel EEG acquisition system will be used (32-channel ZhenTech, China) to record the participants’ brain activity while performing SSRT (Fig. 3). The sampling rate will be set at 1000 Hz, and a notch filter (50 Hz) will be applied to filter out the powerline interference.

Before commencing the experiment, the researcher will brief the participants with the following instructions: (1) ensure minimal movements (except for the hand button movements), especially the head should not sway left or right; (2) refrain from actions such as swallowing and biting while engaging in button pressing; (3) turn off communication devices, such as mobile phones, or set them to silent or in flight mode to mitigate external distractions.

#### Secondary outcomes

##### Box and block test (BBT)

The Box and Block Test (BBT) is a functional test to measure unilateral gross manual dexterity with satisfactory reliability and validity [34], which will serve as the assessment tool to evaluate motor function in stroke patients, including grasping, transporting, and releasing objects. The BBT contains 150 colored wooden cubes, each measuring 1 in. The participants will be tasked with transferring the blocks from one box to another as soon as possible within 60 s, exclusively employing the hemiplegic hand [34]. To facilitate familiarity with the task, participants will be granted a 15-s practice session before the formal assessment commences. Upon the conclusion of the test, the study researcher will count the number of successfully transported blocks. It is important to note that a higher count of transported blocks corresponds to a greater degree of hand function proficiency.

#### Transcranial magnetic stimulation (TMS)

##### Motor-evoked potential (MEP) and central motor conduction time (CMCT)

Single pulse transcranial magnetic stimulation (sTMS, Xiang Yu Medical; China) with a figure-of-eight magnetic coil will be used for measuring motor-evoked potential (MEP) and central motor conduction time (CMCT) [35]. The MEP will be recorded using surface electrodes on the first dorsal interosseous muscle, which is a standard approach for upper limb studies. Participants will be comfortably seated, relaxing their heads and arms. The coil will be placed 5 cm lateral to the vertex along the auricular line (over the M1 of the brain-affected side), aligning at a 45-degree angle from the brain midline, with the handle pointing backwards. The location yielding consistently maximal MEP responses will be identified as the “motor hotspot” [36]. The magnetic stimulus intensity will be set at 20% above the threshold for MEP. The TMS operator will adjust the intensity of the magnetic cortical stimulus to elicit MEPs with a peak-to-peak amplitude of on average 1 mV [37]. Sequential stimulation will be administered five times at each intensity, and the average of the resulting five MEP traces will be used as the outcome for data analysis. Latency will be reported as the mean and standard deviation (SD), while amplitude will be represented by the median due to data distribution skewness.

CMCT denotes the conduction time from the motor cortex to the spinal motor neurons. Spinal magnetic stimulation will be used to measure CMCT. Following the aforementioned cortical stimulation, each participant will undergo cervical stimulation using the coil positioned above the C7 spinous process, situated 2 cm lateral to the midline. The stimulation aims to activate cervical nerve roots at the intervertebral foramina. Participants will encounter an MEP with a latency time attributed to cortical magnetic stimulation of the first dorsal interosseous muscle and find an MEP with another latency time due to cervical magnetic stimulation of the same muscle (peripheral motor conduction time). The difference between the two latency times was the CMCT. They will then experience an MEP characterized by a distinct latency due to cervical magnetic stimulation of the same muscle (representing peripheral motor conduction time). The disparity between these two latency times provides the CMCT value.

##### Short intracortical inhibition (SICI) and intracortical facilitation (ICF)

Paired pulse TMS (pTMS, Xiang Yu Medical; China) will be used to measure short intracortical inhibition (SICI) and intracortical facilitation (ICF), both of which are important indicators for evaluating the plasticity of the cerebral cortex. Building upon the preceding procedures, SICI and ICF entail a stimulation at 80% of the resting threshold (RT), a second stimulation at 120% of RT, and an interstimulus interval (ISI) of 3 ms and 12 ms, respectively [38]. Similar to the MEP assessment, each parameter will be subjected to five stimulations to calculate the average value. All the TMS procedures mentioned above will be completed by a professional physiotherapist with 5 years of clinical experience [39].

##### Magnetic resonance imaging (MRI)

MRI (3-T Siemens Skyra, Erlangen Germany) with a circular surface coil will be used to explore changes in the brain, including gray matter density, cortical thickness, subcortical nuclei volumes, and functional connectivity. Each participant will undergo an MRI scan for approximately 25 min, performed with their eyes closed and extra padding around the ears to reduce noise interference during the MRI scanning process.

Four distinct scanning sequences will be implemented as follows: (1) Resting-state functional MRI images (rs-fMRI): recurrence time (TR) = 2100 ms, echo time (TE) = 30 ms, flip angle = 90°, voxel size = 0.9 isotropic, 42 axial slices, field of view (FOV) = 200 mm × 200 mm, and phases = 230. (2) High-resolution T1-weighted structural images (T1WI): TR = 8.2 ms, TE = 3.2 ms, flip angle = 12°, FOV = 220 mm × 220 mm, matrix = 256,256, slice thickness = 1 mm [40]. (3) Blood oxygenation level-dependent (BOLD) signal: TR = 2000 ms, TE = 30 ms, flip angle = 90°, FOV = 240 mm × 240 mm, thickness = 4 mm, no interval scanning. (4) Diffusion tensor imaging (DTI): TR = 3800 ms, TE = 106 ms, FOV = 230 mm × 230 mm, apply dispersion gradients and 1 no dispersion weight vegetable chicken in 20 directions, where B = 1000 s/mm^2^.

##### RNA-sequencing of the blood sample

Every participant will undergo the collection of a 1-mL blood sample from a vein by a skilled nurse, both at baseline and following 2 weeks of the tACS intervention period. The aim is to discern alterations in neuroinflammatory factors, nerve growth factors, and synaptic plasticity-related genes in the blood milieu [41]. Participants will be instructed to abstain from food consumption after 20:00 the previous evening, along with refraining from drinking and high-intensity exercise prior to the blood draw. Additionally, breakfast consumption will be prohibited on the day of the blood sampling. After emptying the bladder, participants will proceed to a laboratory room. A volume of 1 ml venous blood will be collected into an EDTA anticoagulant tube. To this, 3 ml of TRIzol Reagent (LMAl Bio, China) will be added, ensuring thorough mixing. Subsequently, the mixture will be incubated at 25 °C for a duration of 5 min, after which it will be stored at − 80 °C [41]. This meticulous process facilitates the preservation and stability of the blood samples for subsequent biochemical analysis.

### Participant timeline {13}

The time schedule of enrolment, interventions, assessments, and visits for participants is shown in Table 1.

### Sample size {14}

The sample size calculation was conducted using G*power software (v3.1.9.2). The effect size of this study was estimated from a study conducted by Jaberzadeh et al. who investigated the differential effects of unihemispheric concurrent dual-site and conventional tDCS on motor learning using SRTT [11], which was determined to be 0.526. According to a prior two-way analysis of variance (ANOVA) F test, with a power of 0.95 and an alpha (α) level of 0.05, an estimated 32 participants will be needed. Considering a 20% drop-out rate, the final sample size of each group will be 10, with a total of 39.

### Recruitment {15}

Stroke patients for this study will be recruited from the neurology department of Shanghai Seventh People’s Hospital in China. The recruitment strategy will encompass a variety of methods, including posters, online advertisements, and leaflet distribution. Clear communication regarding the specifics of the experiment will be established with potential participants. Upon the voluntary endorsement of the informed consent by the patients, they will be invited to participate in the study.

## Assignment of interventions: allocation

### Sequence generation {16a}

A total of 39 eligible participants will be assigned to three groups at a 1:1:1 ratio using stratified randomization with sex, age, and stroke severity as factors in this study. The stratified randomization will be achieved as follows: Firstly, participants will be divided into men and women two groups. Secondly, the two groups will be grouped by age (< 60 and ≥ 60). Lastly, the four groups will be grouped by Brunnstrom scale grade (III, IV, V), a total of 12 subgroups. Finally, all 12 groups will be regrouped into three new groups including the SS group, ST group, and MT group to minimize the bias of the final results. The random number will be generated by Microsoft Excel (https://www.microsoft.com) and will be overseen by an external statistician who is not directly involved in the study.

### Concealment mechanism {16b}

Each participant will receive a sealed, opaque envelope containing an assigned random number that determines their respective group assignment.

### Implementation {16c}

The envelopes will be unveiled by an independent researcher only after all participants have concluded the baseline assessments to avoid bias.

## Assignment of interventions: blinding

### Who will be blinded {17a}

It should be noted that complete blinding of both researchers and participants to the allocation of intervention targets is not feasible due to the inherent visibility of stimulation targets. Therefore, blinding will be selectively implemented, extending exclusively to the assessors and statisticians responsible for data collection and final statistical analyses.

### Procedure for unblinding if needed {17b}

Unblinding of group division will occur in situations where the staff deem this necessary for participant safety, to address a technical issue related to the tACS device, or another unforeseen situation in which tACS status is critical for study conduct.

## Data collection and management

### Plans for assessment and collection of outcomes {18a}

The plans for the assessment and collection of outcome, baseline, and other trial data, including any related processes to promote data quality and a description of study instruments along with their reliability and validity, have been described in the “Outcomes {12}” section.

### Plans to promote participant retention and complete follow-up {18b}

Firstly, we will provide the participants with a detailed explanation of the trial, including possible benefits while they sign the informed consent form. Secondly, free examination reports such as fMRI will be provided to participants.

### Data management {19}

As tACS technique is a low-risk intervention which has no substantial safety issues, there will not be a data monitoring committee.

### Confidentiality {27}

All data will be identified and labeled only by subject ID numbers, which will be stored separately from the identifying information and from consent and assent forms.

### Plans for collection, laboratory evaluation, and storage of biological specimens for genetic or molecular analysis in this trial/future use {33}

After the blood samples of the subjects were obtained by professionals, they were thoroughly mixed with TRIzol and stored in a − 80 °C freezer at the central laboratory of Shanghai Seventh People’s Hospital.

## Statistical methods

### Statistical methods for primary and secondary outcomes {20a}

Statistical analysis will be performed using IBM SPSS Statistics 25 (http://www.spss.com.hk). Independent statisticians who remain blinded to the grouping will use an intention-to-treat (ITT) analysis approach to scrutinize the study results. In the case of missing data, the last observation value will be used for interpolation. Continuous variables conforming to a normal distribution will be described as the mean ± SD, while nonnormal distributions will be presented as medians, and the categorical variables will be described using frequency counts. The two-way variance with repeated measures will be performed for continuous variables that meet the assumptions of a normal distribution and homogeneity of variance, while the Wilcoxon test will be used if not. A chi-square test will be performed for categorical variables. The comparison between the three groups will use a two-tailed multivariate analysis of variance. When the *P* value was less than 0.05, the results were statistically significant. The post hoc comparisons will be performed by the Bonferroni correction for multiple comparisons if necessary.

### Interim analyses {21b}

None planned. There are no anticipated problems that are detrimental to the participant.

### Methods for additional analyses (e.g., subgroup analyses) {20b}

None planned.

### Methods in analysis to handle protocol non-adherence and any statistical methods to handle missing data {20c}

The intention-to-treat principle (ITT) will be used to analyze the data.

### Plans to give access to the full protocol, participant-level data, and statistical code {31c}

The plans of this protocol are available on the clinical trial registration website (https://www.chictr.org.cn).

## Oversight and monitoring

### Composition of the coordinating center and trial steering committee {5d}

The principal investigators (PIs) of this clinical trial are PI # 1, Cong Wang, PhD, at the Shanghai Seventh People’s Hospital, Shanghai University of Traditional Chinese Medicine, and PI # 2, Xiaoming Yu, PhD, at the Shanghai Seventh People’s Hospital, Shanghai University of Traditional Chinese Medicine. PI # 1 designs the concept of the research project including the stimulation protocol, the SSRT design, and all the neurophysiological recording methods. PI #1 also performs and analyzes along with her research team all neurophysiological recordings for all study participants of the clinical trial. PI # 2 leads the implementation of multi-target transcranial alternative current stimulation and regular rehabilitation training as well as the clinical assessments for study participants at the hospital. The trial steering committee is composed of the PIs and dedicated research staff.

The PIs and dedicated research staff will form the trial steering committee, which is accountable for coordinating and managing the whole project. Study governance for this single-site study is organized into the oversight team, recruitment team, intervention deployment and assessment team, neurophysiological recording and neuroimaging team, blood sample team, data management, and analysis team. The oversight team is led by the PIs and is responsible for global oversight of the conduct and progress of the study. Each team is led by a dedicated research staff or clinician. Each team works with the oversight team to develop and monitor standard operating procedures. Each team has a weekly meeting with the PI focused on decisions and progress within their scope of responsibility. Full study meetings are held quarterly and as needed.

### Composition of the data monitoring committee, its role, and reporting structure {21a}

Not applicable. As tACS technique is a low-risk intervention which has no substantial safety issues, there will not be a data monitoring committee.

### Adverse event reporting and harms {22}

Due to the tACS technique being a low-risk intervention, no adverse event reporting is anticipated. During the tACS intervention, a professional doctor will always accompany the entire process. If the participant experiences any discomfort, the intervention will be stopped and proper treatment will be provided by the professional.

### Frequency and plans for auditing trial conduct {23}

The Ethics Committee will provide ongoing regulatory monitoring. Planned site visits or the trial dataset exploration will occur, and the trial steering committee and Ethics Committee will meet annually to review the trial conduct upon study initiation yearly.

### Plans for communicating important protocol amendments to relevant parties (e.g., trial participants, ethical committees) {25}

The Ethics Committee of the Shanghai Seventh People’s Hospital needs to review and approve any significant changes to the protocol or informed consent which might cause participant safety risks or impact the scientific soundness of the project.

## Dissemination plans {31a}

We will disseminate the study’s results widely through conference presentations or publications. Publications in high-impact, open-access medical journals and talks at national and international medical conferences will serve this purpose.

## Discussion

The tACS technique has gained increasing attention due to its potential to modulate endogenous neural oscillations through weak electrical currents [42]. The 40-Hz multi-target tACS protocol, grounded in intrinsic brain oscillatory activities, presents a promising and well-tolerated treatment for improving motor learning in stroke patients. The acquisition of novel motor skills, a cornerstone of human behavior, underscores the significance of motor learning [43]. Numerous research articles have confirmed the beneficial impact of tACS, particularly 40 Hz tACS, in exploring memory [44], learning [45], and higher cognitive function [11, 46], all of which are pivotal for effective motor learning. Despite these findings, the impact of 40 Hz tACS on the motor learning of stroke patients is still unclear.

In light of this, we devised three groups—the SS group, the ST group, and the MT group—to explore the therapeutic potential of 40 Hz tACS in stroke patients. This study also seeks to ascertain whether variations in stimulation targets yield divergent treatment outcomes among the three groups. Importantly, we will elucidate the brain mechanism and physiological underpinnings that drive the therapeutic effects through EEG, TMS, MRI, and blood RNA sequencing. In addition, we will assess the improvement in upper limb function in stroke patients using the BBT before and after the 2-week intervention. If 40 Hz tACS proves efficacious, it could become a valuable treatment option for enhancing motor learning in stroke patients.

Nonetheless, it is important to acknowledge certain limitations within the protocol. First, the duration of tACS intervention may not be optimized, and determining the most effective intervention duration remains uncertain. Second, the potential influence of patient-specific characteristics, such as stroke type and location, on treatment outcomes remains unclear. To address these limitations, we intend to conduct further research to refine and optimize the protocol’s outcomes in the future.

## Trial status

Participant recruitment will start in February 2024 and is expected to be completed at the end of April 2024. The version of this protocol is the 2nd version.

## Abbreviations

tACS: Transcranial alternating current stimulation
TMS: Transcranial magnetic stimulation
MRI: Magnetic resonance imaging
SRTT: Series reaction time test
EEG: Electroencephalogram
MEP: Motor evoked potential
CMCT: Central motor conduction time
SICI: Short interval intracortical inhibition
ICF: Intracortical facilitation
BBT: Box and Block test
NIBS: Noninvasive brain stimulation
M1: Primary motor cortex
S1: Primary sensory cortex
DLPFC: Dorsolateral prefrontal cortex
CB: Cerebellar
RT: Rest threshold
ISI: Interstimulus interval
NGF: Nerve growth factor
NAS: Numeric Analog Scales
ITT: Intention to treat
